# Fabrication and Performance of All-Solid-State Chloride Sensors in Synthetic Concrete Pore Solutions

**DOI:** 10.3390/s101110226

**Published:** 2010-11-16

**Authors:** Xiaojian Gao, Jian Zhang, Yingzi Yang, Hongwei Deng

**Affiliations:** School of Civil Engineering, Harbin Institute of Technology, Harbin 150006, China; E-Mails: 395141592@qq.com (J.Z.); yzyang@hit.edu.cn (Y.Y.); denghw200305@yahoo.com.cn (H.D.)

**Keywords:** chloride sensor, all-solid-state, fabrication, potentiometric response, synthetic concrete pore solutions

## Abstract

One type of all-solid-state chloride sensor was fabricated using a MnO_2_ electrode and a Ag/AgCl electrode. The potentiometric response of the sensor to chloride in synthetic concrete pore solutions was systematically studied, and the polarization performance was also evaluated. The results show a good linear relationship between the potential reading of the sensor and the logarithm of chloride activity (concentration ranges from 0.05 to 5.0 M), and the potential value remains stable with increasing immersion time. The existence of K^+^, Ca^2+^, Na^+^ and SO_4_^2−^ ions have little influence on the potentiometric response of the sensor to chloride, but the pH has a significant influence on the potential value of the sensor at low chloride concentration. The potential reading of the sensor increases linearly with the solution temperature over the range from 5 to 45 °C. Meanwhile, an excellent polarization behavior is proven by galvanostatic and potentiodynamic tests. All of the results reveal that the developed sensor has a great potential for monitoring chloride ions in concrete environments.

## 1. Introduction

Chloride-induced corrosion of the steel reinforcements has become the principal cause of premature deterioration of concretes exposed to de-icing salts or marine environments all over the World [1], and it has been estimated that these failures can account for more than 35% of the total volume of construction work in Europe. It is well known that after reaching a threshold concentration, the soluble “free” chloride ions in concrete pore solution result in the passive film collapse and corrosion of steel [2]. Therefore, monitoring of free chloride content is one of the most important methods to prevent and control premature deterioration of concretes in chloride environments. Two types of methods have been used for such a purpose: the leaching method [3], based on the interaction of concrete with water and analysis of the leachate; the pore-pressing technique [4], based on the application of high pressure to a sample of the concrete or mortar allowing extraction of a small portion of liquid that can be analyzed. These two procedures, although well-established, are both destructive and time-consuming, and require periodical sampling from the structure of concern. The use of embeddable sensors for continuous and *in situ* monitoring of chloride content is in its essence a much more straightforward procedure.

Several types of sensors for determining Cl^−^ using different detection principles are reported in the literature. Chloride selective polymeric membrane electrodes are very popular due to their great potential for clinical, environmental and industrial applications [5]. A variety of anion carriers such as Cu(II) complexes [6], and a newly synthesized hydrogen bonding product [7] have been reported to improve the selectivity and sensitivity of this type of sensor to chloride over a wide range of concentrations. Different types of polymeric membranes have also been researched to improve the performance of such sensors [8,9]. However, these sensors have a very narrow working pH range of 4.2–9.6 [6] or 6.5–8.0 [7] and their lifetimes are still in doubt [8,9]. An all-solid-state miniature planar selective chloride electrode was also prepared by using a screen-printing technology, but this electrode had a very short lifetime of only two months [10]. It was demonstrated that Bragg-grating or long period grating-based optical fibre sensors can be applied to chloride ion monitoring in known aqueous solutions [11,12], indicating the possible applications in early stage corrosion monitoring for marine or transport concrete structures. A novel colorimetric sensor for chloride was also developed recently [13], but it is hard to implement for quantitative analysis and field application.

Ag/AgCl electrodes are stable, easily prepared, and have long been used in the electrochemistry field as chloride ion selective or reference electrodes [14]. Their equilibrium potential value depends on the chloride ion activity (concentration) of the surrounding solution according to Nernst’s Law. The use of Ag/AgCl electrodes to determine chloride activity in simulated cement pore solution, in pressure-extracted cement pore water and even in mortar and concrete samples has been described in the literature [15–17]. The Ag/AgCl wire has even been used as a sensor for determination of water soluble chloride in admixtures and aggregates for cement [18]. Atkins *et al.* [19] discussed the effect of temperature and the presence of bromides in seawater on the potential readings of Ag/AgCl electrodes. In spite of the doubts about the long-term stability risen in literature [16], Ag/AgCl electrodes still remain a potentially interesting choice in what concerns chloride ion monitoring in reinforced concrete structures [20]. At the same time, all the experiments in literatures were carried out by using saturated calomel or saturated Hg_2_SO_4_ electrodes as references. These reference electrodes are unsuitable for *in situ* monitoring because the liquid electrolytes in them may leak or become invalid if exposed to cold temperatures. Several solid electrodes such as alkaline manganese dioxide (MnO_2_), metal-metal oxide (MMO) and graphite electrodes may be suitable for embeddable use in concrete environments [21]. The electrochemical properties and long-term stability of MnO_2_ electrode have been extensively studied in saturated calcium hydroxide solution, concrete pore solution and cement extracts [22–24]. It was found that the MnO_2_ electrode has long-term stability in concrete environments and even the addition of chloride has little influence on its performance. One embeddable MnO_2_ reference electrode has been developed for measuring the potential of steel rebar to evaluate its corrosion status [25].

This paper aimed at fabricating one type of all-solid-state embeddable chloride sensor using a Ag/AgCl electrode as working electrode and a MnO_2_ electrode as reference, which can indicate chloride activity through its potential reading without the need for any additional reference electrodes. The performance of such a chloride sensor were extensively evaluated using synthetic concrete pore solutions.

## 2. Experimental

### 2.1. Raw Materials

Ordinary Portland cement (OPC) conforming to GB175-2007 was used. Polyvinyl alcohol (PVA) fiber with a diameter of 39 μm and length of 3–5 mm was used to reinforce the cement paste. A MgO-based expansive agent was used to compensate for cement shrinkage during hydration. Manganese dioxide and acetylene carbon black (reagent grade ≥92% purity) were used for making the reference electrode cell; Ag and AgCl powder (reagent grade ≥99.9% purity) were used for making the working electrode. Analytical-reagent grade NaOH, KOH, CaO and NaCl were selected. Distilled deionized water was used to prepare all aqueous solutions.

### 2.2. Fabrication of the Chloride Sensor

**Figure 1.**
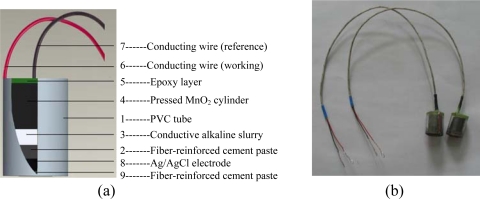
**(a)** Schematic layout of the chloride sensor structure. **(b)** Photograph of the completed chloride sensor.

As shown in Figure 1(a), this chloride sensor is a PVC tube with a multilayer filling. The fabrication process of the sensor can be briefly described as follows:

1. A suitable PVC tube with a diameter of 10 mm and length of 15 mm was chosen. This tube should be strong and impermeable enough to protect the sensor from any mechanical damage or chemical corrosion during the test.
2. Cement, PVA fiber and MgO-based expansive agent were mixed with water to prepare a fiber-reinforced cement paste. Proper mix proportioning was performed to minimize the risk of shrinkage cracking. About 2–3 mm depth of such cement paste was applied in the tube with a 3–4 mm distance from the bottom. This cement paste layer had a very low permeability and improved cracking tendency, therefore, the leakage of the alkali from the top of this layer can be diminished markedly. On the other hand, this paste layer acts as the electrical contact media for the top (reference electrode) and the bottom (working electrode) parts of the sensor.
3. A special alkaline slurry composed of sodium, potassium and calcium hydroxides was placed on top of the cement paste after several days of wet curing. The pH of the slurry was equal to that of the cement paste. This slurry was used to supply a constant alkaline environment (pH = 13.5) and the leakage of alkaline solution can thus be avoided.
4. MnO_2_ powder was mixed with a known quantity of acetylene carbon black, which is used to improve the electrical contact among MnO_2_ particles, and then compressed into a cylindrical shape with a specially designed steel mould. The MnO_2_ cylinder was implanted with a low resistance conducting wire during the preparation. It was finally placed into the tube from the top with a controlled pressure to ensure tight contact between the cement paste, alkaline gel and MnO_2_ cylinder.
5. The top of the tube was sealed with epoxy to complete the MnO_2_ reference electrode. After the hardening of the epoxy, the MnO_2_ sensor electrode was activated with synthetic concrete pore solution (0.2 M NaOH + 0.6 M KOH + saturated Ca(OH)_2_ solution) for a few days. The half-cell potential of MnO_2_ electrode is determined by MnO_2_/Mn_2_O_3_ equilibrium potential [24]. In this study, the compressed MnO_2_ was surrounded by the slurry with a stable alkalinity of pH 13.5. Therefore, the activated MnO_2_ electrode kept a stable potential and was used as the reference electrode.
6. A mixture of Ag and AgCl powder with an optimal proportion was compressed into a tablet shape with a height of about 1.5 mm and diameter of less than the PVC tube. The Ag/AgCl tablet, as the working electrode (chloride ion selective electrode), was placed at the bottom of the above mentioned tube assembled with MnO_2_ electrode and then installed and encapsulated with a fiber-reinforced cement paste with a higher porosity than the one mentioned above.

Completed all-solid-state chloride sensors are shown in Figure 1(b). When exposed to a chloride environment with temperature *T* and chloride ion activity of *α*_*Cl*^−^_, the potential of Ag/AgCl electrode *E_Ag/AgCl_* is given by the Nernst equation:

(1) $$E_{\mathrm{Ag}/\mathrm{AgCl}}=E_{\mathrm{Ag}/\mathrm{AgCl}}^{0}-\frac{\mathrm{RT}}{F}\text{log}\;\alpha_{{\mathrm{Cl}}^{-}}$$

where $E_{\mathrm{Ag}/\mathrm{AgCl}}^{0}$ stands for the standard electrode potential of the Ag/AgCl electrode, R for the universal perfect gas constant (8.3145 J·mol^−1^·K^−1^), T for absolute temperature (K), F for Faraday constant (96,485 C·mol^−1^).

The MnO_2_ electrode acts as the reference electrode (with the potential of *E*_ref_) in this study, and the sensor potential can be expressed as:

(2) $$E=E_{\mathrm{Ag}/\mathrm{AgCl}}-E_{\mathrm{ref}}=(E_{\mathrm{Ag}/\mathrm{AgCl}}^{0}-E_{\mathrm{ref}})-\frac{\mathrm{RT}}{F}\text{log}\;\alpha_{{\mathrm{Cl}}^{-}}$$

The potential *E* can be measured through two conducting wires of the sensor, and then the chloride activity can be determined according to the calibrated curve.

### 2.3. Measurement Methods

1. Potentiometric response and stability: The potential of the sensor was tested in synthetic concrete pore solutions with different concentrations of NaCl ranging from 0.05 to 5.0 M. The working stability was assessed by measuring the potential development during 60 days of immersion in synthetic concrete pore solution. Two parallel sensors were measured in these tests.
2. Influences of other ions and pH on potentiometric response to chloride: Ions such as Ca^2+^, K^+^, Na^+^, SO_4_^2−^ may exist in concrete in different concentrations depending on the raw materials used and service environments such as marine, de-icing salts and sulfate attack [26]. For the fully carbonated concretes, the pH value of the pore solution decreases to as low as 7.5 [27]. Therefore, four series solutions were prepared: distilled water (O), saturated Ca(OH)_2_ solution (A), synthetic concrete pore solution (B) and synthetic concrete pore solution plus 0.1 M concentration of SO_4_^2−^ (C), and then 0.1 M and 1.0 M concentrations of chloride ions were added using NaCl, KCl and CaCl_2_ respectively. Potential readings of the sensor in these solutions were tested.
3. Influence of temperature: When the solution temperature ranged from 5 °C to 60 °C, the potentiometric responses of the sensor to two chloride concentrations of 0.1 and 1.0 M were measured.
4. Polarization performance: Galvanostatic and potentiodynamic polarizations were performed on the chloride sensor in synthetic concrete pore solutions with 0.1 and 1.0 M concentrations of NaCl. Anodic and cathodic direct currents of 10 μA were applied to the sensor for seven hours. A highly-purified titanium plate with an area 10 times greater than the cross section of the sensor tube was used as a counter electrode, and Ag/AgCl and MnO_2_ cells of the sensor were used as working electrode and reference electrode respectively. In potentiodynamic measurement, a potential range of −100–300 mV was used for 0.1 M chloride solution and −150–250 mV for 1.0 M solution, with a sweep rate of 1 mVs^−1^.

## 3. Results and Discussion

### 3.1. Potentiometric Response to Chloride Concentration

The uniformity of chloride sensor was evaluated in synthetic concrete pore solution with a chloride concentration of 0.1 M. Potentiometric response was measured for each of twenty chloride sensors. The potential values of these sensors range from 112 to 119 mV, with the maximum variation of only ±3.5 mV. The manufacturing procedure of the Ag/AgCl electrode is very well-established, therefore the potential variation is as low as ±1.0 mV when exposed to a wide range of chloride concentrations (10^−3^–1.0 M) [22]. The potential variation should be mainly attributed to the MnO_2_ reference electrode. Muralidharan [23,25] reported the alkaline solid MnO_2_ electrode with the maximum variation of ±5 mV *versus* SCE when exposed to saturated calcium hydroxide solution. The improved variation in potential for chloride sensors in this study is due to the better impermeability of the fiber-reinforced cement paste and the special structure eliminating the alkali leakage from the MnO_2_ electrode cell. This difference in potential reading seemed negligible indicating the uniformity of chloride sensors prepared with this procedure as described in Section 2.2. However, each chloride sensor should be calibrated separately before measurement due to even a few mV strongly affecting the measuring accuracy of chloride ion concentration [28].

**Figure 2.**
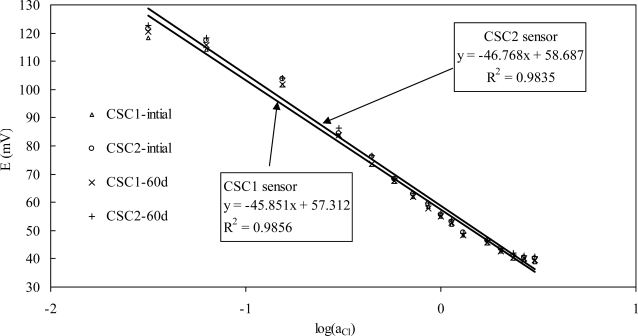
Potentiometric response of the sensor to chloride activity.

The potentiometric responses of two parallel sensors (CSC1 and CSC2) to chloride ion activities in synthetic concrete pore solutions are presented in Figure 2. The activity coefficient of Cl^−^ at different concentration solutions were determined according to Vera’s work [29] on the basis of the Pitzer approach [30], which is the first model to consider the association of Cl^−^ ions with alkali and alkali-earth metal ions and has been proven to be suitable for the alkaline environments. For every chloride ion activity (concentration), a very limited difference in potential reading was observed between the two sensors: a maximum of 3.2 mV for 0.05 M concentration and 1.2 mV for 5.0 M concentration. For each sensor, the potential reading decreases with increasing chloride ion concentration ranging from 0.05 to 5.0 M, and a good linear relationship (with a correlation coefficient of higher than 0.98) is observed between the potential reading and the logarithm of chloride ion activity as described by the Nernst equation. However, a slight bigger deviation from linearity can be found at low Cl^−^ concentrations, as reported by Miguel [16]. Therefore, such sensors showed a lower sensitivity at low chloride concentrations which are not very important in most cases for embeddable monitoring of steel-reinforced concrete. The Ag/AgCl electrode can maintain a stable potential (±2 mV deviation) when embedded in the concrete containing higher than 0.4% of cement weight [16] or exposed to simulated concrete pore solution for 40 days [22]. It was reported that the potential *versus* SCE of MnO_2_ electrode was increased by 6 mV after 90 days exposure to synthetic concrete pore solution [31]. For the chloride sensor assembled by an Ag/AgCl electrode and a MnO_2_ electrode, a better potential stability was found in Figure 2. After 60 days of exposure to synthetic concrete pore solution, the potential reading difference from the initial value is less than 2.2 mV for all the solutions. The improved potential stability of the sensor should be attributed to the better performance of the MnO_2_ electrode which is encapsulated in the top part of sensor, indicating the possible application for long-term monitoring in concrete environment.

### 3.2. Influences of Other Ions on Potentiometric Response

**Figure 3.**
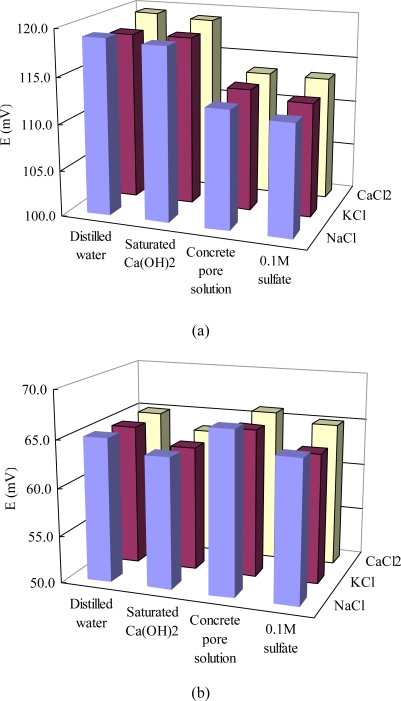
**(a)** Potentiometric responses of the sensor in different chloride solutions with 0.1 mol/L concentration of Cl^−^. **(b)** Potentiometric responses of the sensor in different chloride solutions with 1.0 mol/L concentration of Cl^−^.

Figure 3 presents potential readings of the sensor in chloride solutions containing other ions. Every solution sample was tested six times with the same sensor and the average value was obtained. The potential values showed a very low standard deviation (less than ±0.7 mV) for each solution. Under the same chloride ion concentration of 0.1 M, the sensor potentials in solutions based on distilled water (O) and saturated Ca(OH)_2_ solution (A) presented 5.0–6.3 mV higher than those in solutions based on synthetic concrete pore solution (B). This decreased potential values in synthetic concrete pore solution, which has a notably higher pH value (13.5) than distilled water (7.0) and saturated Ca(OH)_2_ solution (12.0), is mainly due to the interference from hydroxide (OH^−^) ions. This interference has seldom been quantified before for the case of concrete pore solution, but abnormally higher Cl^−^ activities (lower potential) were obtained potentiometrically at low Cl^−^ concentration solutions (below 0.1 M) [29,31]. This deviation at low chloride contents could be attributed to the formation of AgOH [32]. No visible difference (less than 1.0 mV) in potentials were found before and after the addition of 0.1M SO_4_^2−^ in solutions B. When the chloride ion concentration increased to 1.0 M, the potential of the sensor in saturated Ca(OH)_2_ solutions was a little lower (1.4–1.7 mV) than those in distilled water solutions. However, the potentials in synthetic concrete pore solutions were (0.7–1.8 mV) higher than those in distilled water solutions. After the addition of sulfate, the potential was increased by 0.9–2.0 mV. Therefore, the influences of pH and sulfate on the potential of the sensor in 1.0 M chloride solutions is different from those in 0.1 M chloride solutions, but the potential variation is less significant in 1.0 M chloride solution. For both 0.1 and 1.0 M chloride concentrations, different cations (Ca^2+^, K^+^, Na^+^) have very limited influence on potentiometric response to chloride (average less than 1.0 mV), indicating such sensors can most likely be applied to a wide range of concrete environments exposed to different chlorides. The pH value of a fresh concrete may be as high as 13.5. As carbonation proceeds, the pH value of the concrete pore solution decreases to as low as 7.5 [27]. According to the above results, the pH value should be simultaneously detected in order to precisely determine the chloride content in chloride-slightly contaminated concretes. Further studies should be carried out to quantify the interference of pH and found out a suitable calibration method for low chloride concentration environments. Bromide ion, which normally exists in the seawater, was also found to have a significant increasing influence on the measured chloride concentration [19]. To solve this problem, Muguruma [33] developed a thin, nanoporous, plasma-polymerized coating of hexamethyldisiloxane on an Ag/AgCl electrode.

### 3.3. Influence of Temperature on Potentiometric Response

For temperature *T* and chloride ion activity of *α*_*Cl*^−^_, Equation (2) can be expressed in another way:

(3) $$E=(E_{\mathrm{Ag}/\mathrm{AgCl}}^{0}-E_{\mathrm{ref}})-\frac{\mathrm{RT}}{F}\text{log}\;\alpha_{{\mathrm{Cl}}^{-}}=-\frac{R\;\text{log}\;\alpha_{{\mathrm{Cl}}^{-}}}{F}T+(E_{\mathrm{Ag}/\mathrm{AgCl}}^{0}-E_{\mathrm{ref}})$$

If the potential of the reference electrode is constant, there is a good linear relationship between temperature and the potential value of the sensor. The theoretical slopes are 0.1036 and 0.0204 for 0.1 M and 1.0 M chloride concentrations respectively. But there is no evidence that the potential of MnO_2_ keeps stable with the temperature, and further studies should be done in this field.

**Figure 4.**
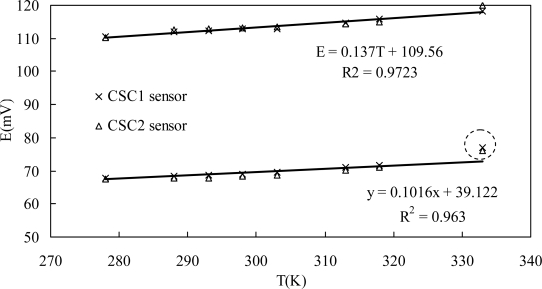
Influence of solution temperature on potentiometric response of the sensor to chloride.

Figure 4 shows the potential readings of two parallel sensors (CSC1 and CSC2) in two chloride solutions with temperatures ranging from 5 to 60 °C. These two parallel sensors showed almost the same values, with a maximum difference of 1.6 mV. For both 0.1 M and 1.0 M chloride concentrations, the relationship between potential output of the sensor and the solution temperature can be expressed by a good linear equation as shown in Figure 4 Montemor [17] found similar influences of temperature on potential values of the Ag/AgCl electrode *vs*. calomel reference electrode. However, the slopes of the regressed lines in this study are very different from the theoretical slopes, this being partially attributed to the influence of temperature on the MnO_2_ electrode. On the other hand, the measured potential at 60 °C was very far from the regressed linear relationship for 1.0 M chloride concentration. Therefore, the potential of the developed chloride sensor can be compensated with such linear regressed equations when the temperature ranges from 5 to 45 °C. However, concrete structures in service maybe undergo more complicated climate change such as freeze-thaw cycles or iterative temperature changes [34], performances of the sensor under such conditions will be further investigated.

### 3.4. Galvanostatic Test

**Figure 5.**
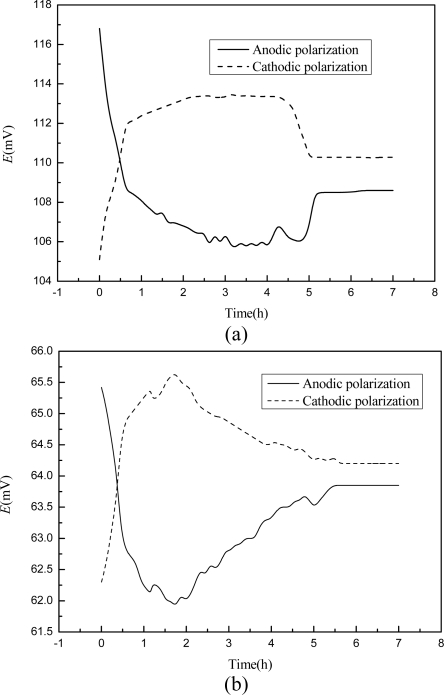
**(a)** Galvanostatic polarization curve of the sensor in synthetic concrete pore solutions with 0.1 mol/L NaCl. **(b)** Galvanostatic polarization curve of the sensor in synthetic concrete pore solutions with 1.0 mol/L NaCl.

Steel corrosion in concrete is normally an electrochemical reaction process [35] and concrete structures may be exposed to stray currents coming from railways, cathodic protection systems, or high voltage power lines [36]. A small amount of current is sometimes involved in the corrosion monitoring application of embeddable sensors [24]. As has been shown in literature [19], such electric fields maybe induce a potential shift in electrodes. Therefore, the polarization of this developed chloride sensor should be tested to evaluate its possible embeddable application in concrete structures. The galvanostatic test was carried out three times for each chloride solution and the results were very close. The representative potential development of chloride sensor under external constant current is presented in Figure 5. For both chloride concentrations, the potential output was very high and very low at the start instant of anodic and cathodic polarizations respectively, and went back to the relative stable value within less than 0.5 hour. In 0.1 M chloride solution, the sensor reading recovered to very stable values of 110.3 mV and 108.5 mV after 5.5 hours of anodic and cathodic polarizations respectively, leading to 5 to 7 mV lower than that before polarization. In 1.0 M chloride solution, the sensor reading recovered to very stable values of 64.2 mV and 63.7 mV after 6 hours of anodic and cathodic polarizations respectively, leading to 3 to 4 mV lower than that before polarization. After the interruption of the current, the potential of the sensor recovered almost to the original value (±1.2 mV for 0.1 M solution and ±0.7 mV for 1.0 M solution) in 2 hours. This potential recovery performance of Ag/AgCl electrode was also reported by Duffó when several electrodes were characterized to be used as reference electrodes embedded in concrete structures [22].

### 3.5. Potentiodynamic Polarization

**Figure 6.**
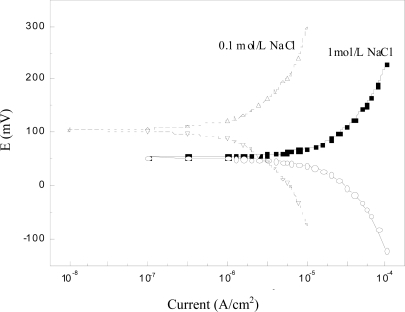
Potentiodynamic polarization curves of sensor in synthetic concrete pore solutions with chloride.

Results of the potentiodynamic polarization tests are shown in Figure 6. It was observed that the chloride sensor showed very negligible current lower than the order of nA/cm^2^ to μA/cm^2^ in both high alkaline concrete pore solutions with chlorides, indicating that it is able to withstand small currents with a minimum of polarization. Further, no visible variation was noticed either in the cathodic or in the anodic polarization curves. In addition, a stable potential passive region was observed in the anodic direction, in accordance with the results of MnO_2_ electrode studied by other researchers [24]. When exposed to pH 13.5 solutions, the Ag/AgCl electrode (prepared by an anodized Ag wire) presented an anodic peak at low overpotentials, followed by a region of constant current densities in the potentiodynamic polarization curve [22]. This anodic peak is probably due to the formation of some silver oxides. No such phenomenon was found in this study, being attributable to the better stability of the Ag/AgCl electrode prepared by the compressed Ag/AgCl powder as described in Section 2.1. Even a small current is induced during polarization, the potentiometric response of the sensor to chloride not changed. Therefore, this behavior is quite suited for the interest to choose it as an embeddable and *in-situ* monitoring sensor for concrete environments.

## 4. Conclusions

The following conclusions can be drawn from the present investigation:

1. An all-solid-state chloride sensor was fabricated using a MnO_2_ electrode and a Ag/AgCl electrode, and a good linear relationship exists between the potential reading of the sensor and the logarithm of chloride activity (concentrations of 0.05–5.0 M) in high-alkaline simulated concrete pore solutions. This sensor has good stability of potentiometric response to chloride with increasing time.
2. The existence of K^+^, Ca^2+^, Na^+^, SO_4_^2−^ ions has very little influence on the potentiometric response of the sensor to chloride. Meanwhile, the potential reading of the sensor increases linearly with the solution temperature (5–45 °C). When the temperature increases to 60 °C, the potential of the sensor deviates from the linearity relationship.
3. The sensor shows an excellent polarization performance under both galvanostatic and potentiodynamic measurements. Therefore, such chloride sensors have great potential to be applied for embeddable and *in situ* monitoring of chloride content in concrete structures.
4. It is suggested that the pH value should be simultaneously detected in order to precisely determine the chloride content in chloride-slightly contaminated concretes. The performances of the chloride sensor embedded in real concrete samples are being extensively studied and more information will gained to support the possible field application.
